# Correlation analysis of *Helicobacter pylori* infection and pathological changes of gastric mucosa in the population of Jiuquan area, China, and Logistic regression analysis of lesion risk factors

**DOI:** 10.3389/fcimb.2026.1827080

**Published:** 2026-07-14

**Authors:** Bing Li, Li Zhen, Junwei Wang, Yonglin Chen

**Affiliations:** 1 The 1 School of Clinical Medicine, Lanzhou University, Lanzhou, Gansu, China; 2 Department of Pathology, Tianjin Huayinkang Medical Testing Laboratory Co., Ltd., Tianjin, China; 3 Department of Pathology, Shanghai General Hospital Jiuquan Hospital, Jiuquan, Gansu, China; 4 Department of Pathology, The First Hospital of Lanzhou University, Lanzhou, Gansu, China

**Keywords:** correlation analysis, gastric mucosa, *Helicobacter pylori*, Jiuquan, China, logistic regression analysis, risk factors of lesions

## Abstract

**Objective:**

To investigate the correlation between *Helicobacter pylori* (H. *pylori*) infection and pathological changes in the gastric mucosa (GM), and to perform logistic regression analysis (LRA) to identify independent risk factors for gastric mucosal lesions (GML).

**Methods:**

A total of 800 individuals with H. *pylori*-positive chronic gastritis (CG) were enrolled as the case group, and another 800 individuals with H. *pylori*-negative CG were selected as the control group. The dataset was randomly divided into a training cohort and a validation cohort at a ratio of 7:3.

**Result:**

Compared with the control group, the H. *pylori*-positive group had significantly higher proportions of individuals aged ≥50 years, those working as farmers or manual laborers, and those who frequently consumed unboiled water. Pearson correlation analysis revealed significant correlations between H. *pylori* infection and various GML. Multivariate logistic regression analysis identified the following independent risk factors for H. *pylori* infection: age ≥50 years, occupation as a farmer or manual laborer, frequent consumption of unboiled water, consumption of grilled foods, habit of sharing meals, and disease duration >2 years. Conversely, tea consumption was significantly associated with a reduced risk of infection, indicating a potential protective effect. A risk prediction model was constructed, and the model demonstrated high discrimination with sensitivities of 88.59% and 82.06%, and specificities of 81.17% and 78.96% in the training and validation sets, respectively.

**Conclusion:**

In the Jiuquan population, H. *pylori* infection is significantly correlated with an increased severity of GML. Key independent risk factors comprise older age, occupational exposure, and poor dietary and hygiene behaviors.

## Introduction

Globally, *Helicobacter pylori (*H. *pylori)*, a Gram-negative microaerobic bacteria, is the primary cause of stomach cancer, chronic gastritis (CG), and peptic ulcers (Graham, 2024). The World Health Organization (WHO) has designated H. *pylori* as a Group 1 carcinogen. Nearly half of the people on the planet suffer from H. pylori infections, with prevalence rates in several poor nations surpassing 70% (Chey et al., 2024). China, bearing a substantial burden of H. *pylori* infection, exhibits significant regional variation in prevalence. In particular, the northwestern region has sustained a high infection rate over time, attributed to factors such as geographic conditions, dietary practices, and suboptimal hygiene (Ren et al., 2022). Gansu Province, a multi-ethnic region in northwest China, reports H. *pylori* infection rates ranging from 50% to 70%, and the incidence of gastric cancer ranks among the highest nationwide (Sonnenberg, 2022).

Situated in northwestern Gansu, Jiuquan features an arid climate and limited water resources. Local dietary patterns predominantly emphasize high-salt foods and grilled items, while certain rural communities retain practices such as consuming unboiled water and communal dining—factors that may heighten H.*pylori* transmission risks (Zhou et al., 2024). Furthermore, underdeveloped sanitation infrastructure, coupled with suboptimal oral and household hygiene conditions, creates an environment conducive to bacterial colonization and dissemination.

At present, the association involving H. *pylori* infection and pathological changes of gastric mucosa (GM) has been confirmed by many studies (Shuman et al., 2024). Long-term infection results in CG and gradually progress to precancerous lesions such as gastric mucosal atrophy, intestinal metaplasia, and dysplasia, ultimately increasing the risk of gastric cancer (Wang et al., 2022; Wessler and Posselt, 2023). Nevertheless, most previous studies have focused predominantly on eastern coastal or economically developed regions of China, whereas research specifically targeting inland northwestern areas such as Jiuquan remains relatively limited. Regional variations in dietary patterns, lifestyle behaviors, and environmental exposures may exert a significant influence on the epidemiological features and pathogenic mechanisms of H. *pylori* infection. Therefore, region-specific investigations are essential to better understand the local transmission dynamics and to inform targeted prevention and control strategies (Sonnenberg, 2022). Furthermore, several studies have investigated risk factors related to *H. pylori* infection and its clinical outcomes (Demirci et al., 2022; Tran et al., 2022), there remains a need to investigate region-specific risk factors and their associations with the severity of gastric mucosal lesions (GML) in the Jiuquan area. To address this gap, the present study focused on patients with CG in Jiuquan, China. Utilizing a case-control study design, the correlation involving H. *pylori* infection and pathological changes in the GM was systematically evaluated. To find independent risk variables linked to H. *pylori* infection in the local community, a logistic regression model was utilized. The goal was to offer a scientific basis for creating preventative and control plans tailored to a particular area (Deng et al., 2022; Chen et al., 2024). Additionally, this study helps bridge the research gap in northwestern China and contributes to the early detection and personalized management of gastric precancerous lesions.

## Materials and methods

### Study the flowchart

**Figure 1:**
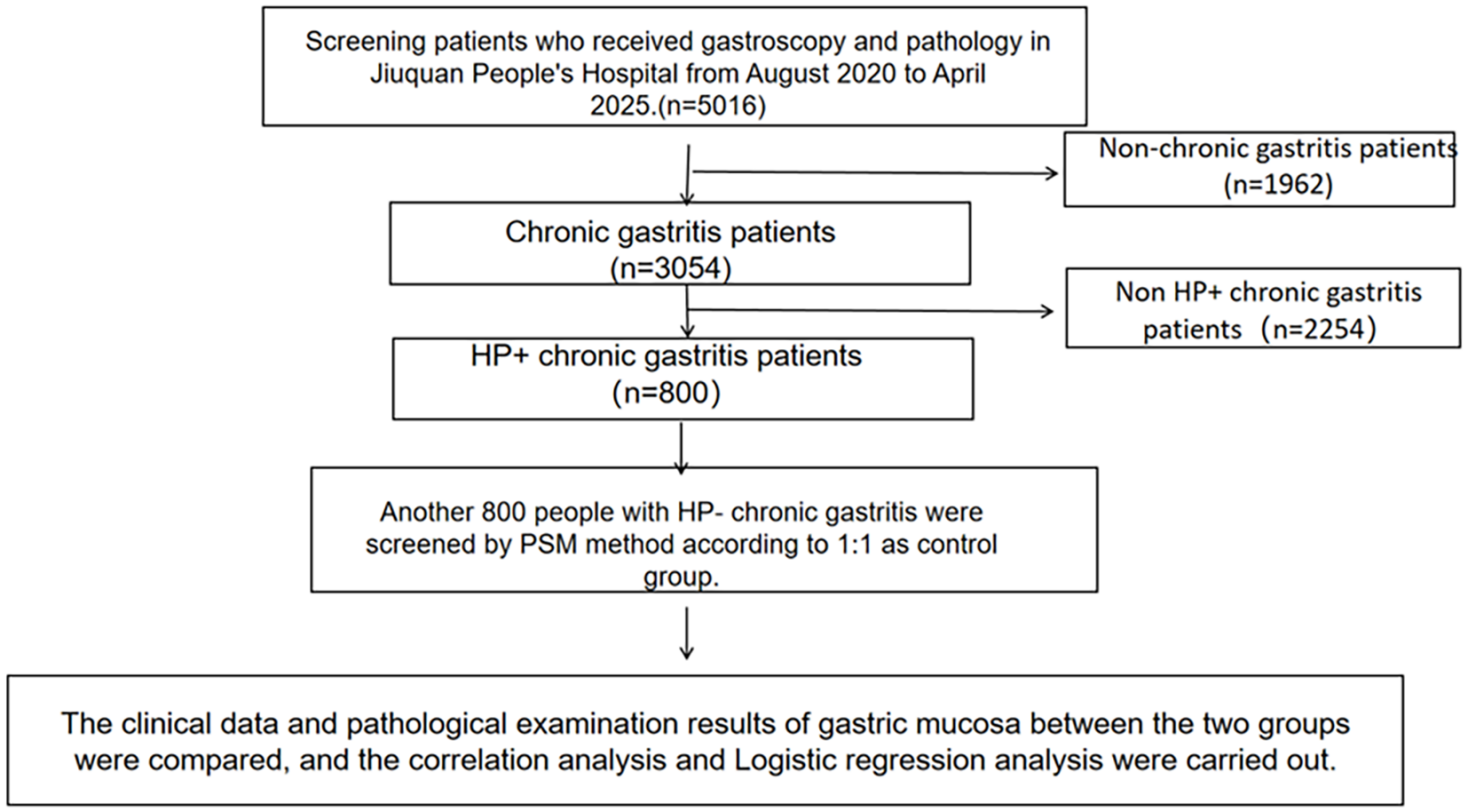
Research flowchart.

The flowchart illustrates the patient selection process, exclusion criteria, and grouping strategy in this study. See **Figure 1**.

### Clinical data

#### Subjects

A retrospective observational study design was employed. A total of 5,016 patients who underwent gastroscopy and pathological examination at Shanghai General Hospital Jiuquan Hospital between August 2020 and April 2025 were screened. Among them, 3,054 patients were diagnosed with CG. Of these, 800 individuals with H. pylori-positive CG were included as the H. pylori-positive group. Using 1:1 frequency matching for age (± 5 years), sex, ethnicity, and residence, an additional 800 individuals with H. pylori-negative CG were selected as the control group. Baseline demographic and clinical characteristics of the two groups are summarized in **Table 1**. Differences between groups were assessed by chi-square tests, and variables with P < 0.05 were considered as potential confounders for subsequent logistic regression analysis. Baseline demographic and clinical characteristics of the two groupings are summarized in **Table 1**. There were no discernible variations between the groups with regard to gender, ethnicity, alcohol use, or smoking status (*P* > 0.05). This study was not designed as a case-control study, but rather a retrospective matched observational analysis based on hospital electronic medical records.

**Table 1:** Baseline characteristics comparison ( $\bar{x}$ ± s, n/%).

Characteristics	Case group (n=800)	Control group (n=800)	*X^2^ value*	*P* value
Basic demographic information				
Gender (Male, %)	435 (54.38)	429 (53.63)	0.091	0.763
Age (≥50 years old, %)	480 (60.00)	363 (45.38)	34.322	<0.001
Ethnicity (Han, %)	793 (99.13)	795 (99.38)	0.336	0.562
Occupation (farmer, worker, %)	461 (57.63)	327 (40.88)	44.900	<0.001
Educational level (high school and below, %)	453 (56.63)	448 (56.00)	0.064	0.801
Residence (Rural area), %)	634 (79.25)	637 (79.63)	0.034	0.853
Living habits				
Consumption of unboiled water (Yes, %)	637 (79.63)	203 (25.38)	472.070	<0.001
Consumption of spicy and stimulating food (Yes, %)	549 (68.63)	561 (70.13)	0.424	0.515
Consumption of grilled foods (Yes, %)	593 (74.13)	327 (40.88)	180.962	<0.001
Tea consumption (Yes, %)	228 (28.50)	582 (72.75)	313.339	<0.001
Smoking (Yes, %)	432 (54.00)	445 (55.63)	0.427	0.514
Alcohol consumption (Yes, %)	451 (56.38)	448 (56.00)	0.017	0.896
Habit of sharing meals (Yes, %)	527 (65.88)	204 (25.50)	262.777	<0.001
Contact with animals (Yes, %)	453 (56.63)	449 (56.13)	0.041	0.840
Disease course (>2 years, %)	528 (66.00)	262 (32.75)	176.918	<0.001
Disease-related indicators				
Hemoglobin (<120 g/L, %)	312 (39.00)	179 (22.38)	51.977	<0.001
Albumin level (<35 g/L, %)	277 (34.63)	153 (19.13)	48.900	<0.001
Previous history of peptic ulcer (Yes, %)	204 (25.50)	133 (16.63)	18.950	<0.001
Family history of tumors (Yes, %)	173 (21.63)	52 (6.50)	75.719	<0.001

All subjects provided written informed permission, and the study was approved by the Ethics Committee of Shanghai General Hospital Jiuquan Hospital (Approval No. 20250605027; approval date: June 5, 2025).

Inclusion criteria: (1) Age ≥18 years and long-term residence in the Jiuquan area (within 5 years); (2) Previously diagnosed with CG in outpatient or inpatient settings (pathological classification based on the *Consensus Opinion on CG in China* (Demirci et al., 2022)), with subsequent confirmation of H. *pylori* infection by gastroscopy and histopathological biopsy; (3) Complete clinical data available; (4) The patient or their legal representative must provide written informed consent.

Exclusion criteria: (1) History of H. *pylori* eradication therapy or use of antibiotics, proton pump inhibitors (PPIs), or bismuth-containing agents for ≥2 weeks within the past 6 months; (2) History of gastric cancer, gastrectomy, severe cardiac, hepatic, or renal diseases, or immunodeficiency disorders (e.g., HIV); (3) Pregnant or lactation; (4) Incomplete medical records or refusal to cooperate with data collection; (5) Any other condition deemed by the investigators to interfere with participation in the study.

### Methods

#### Collection of basic clinical information

Data collection encompassed the following variables: demographic characteristics (age, sex, occupation, ethnicity, place of residence, educational level, and disease duration); lifestyle factors (dietary patterns, communal eating habits, drinking water sources, and specific dietary preferences, including frequent consumption of grilled foods, spicy or irritant foods, and tea consumption); and behavioral factors (smoking, alcohol consumption, and contact with domestic or wild animals).

#### *H. pylori* detection

Detection of H. *pylori* Infection: Immunohistochemical staining is employed for the detection, with the result interpretation criteria as follows:

1. HP(-): In the gastric mucosal tissue section, no H. *pylori* bacteria stained by specific antibodies are observed.
2. HP(+): Under the microscope, scattered or focal small numbers of H. *pylori* bacteria stained by the dye can be clearly identified (the number of bacteria is small, and they may only be present in individual glands or a small localized area).
3. HP(++): Under the microscope (at high - power field), a moderate number of H. *pylori* bacteria stained by the dye are observed (the bacteria are distributed relatively widely, continuously visible in multiple glands or a large area of the mucosal region, but have not yet formed dense colonies covering the area).
4. HP(+++): Under the microscope (at high - power field), a large number and dense clustering of H. *pylori* bacteria stained by the dye are observed (the bacteria are often distributed in clusters or patches, covering a wide area of the mucosa and forming a distinct bacterial layer or colonies in the gastric pits and mucous layer).

#### Pathological examination of gastric mucosa

Gastric mucosal biopsy specimens were obtained during gastroscopic examination and included, but were not limited to, tissues from the gastric antrum, body, angularis incisura, fundus, anterior wall of the antrum, lesser curvature of the gastric body, and any observed gastric polyps. Histopathological analysis was subsequently performed on the collected specimens. The following parameters were evaluated: (1) Activity of Inflammation: Graded as inactive (0), mild (+), moderate (++), or severe (+++); (2) Types of lesions: Included chronic inflammation, atrophic changes, intestinal metaplasia, polypoid hyperplasia, dysplasia/intraepithelial neoplasia.

### Statistical analysis

SPSS 21.0 was used for statistical analysis. Continuous data were first tested for homogeneity of variance and normality. Data following a normal or near-normal distribution were expressed as mean ± standard deviation ( $\bar{x}$ ± s), and the independent-samples t-test was used for two-group comparisons. Categorical data were presented as numbers and percentages [n (%)] and analyzed using the chi-square (χ²) test. Pearson correlation analysis was employed to examine associations between variables. Logistic regression analysis (LRA) was performed to identify risk factors for H. pylori infection in patients with CG.

The logistic regression model was expressed as:


$$\text{logit}\;\left(\text{P}\right)\;=\;{\text{β}}_{0}\;+\;{\text{β}}_{1}{\text{X}}_{1}+{\text{β}}_{2}{\text{X}}_{2}+{\text{β}}_{3}{\text{X}}_{3}+\cdots \cdots +{\text{β}}_{10}{\text{X}}_{10}$$

where:

• PPP: probability of H. pylori infection
• β_0_: intercept
• X_1_: age (≥50 years = 1, <50 years = 0)
• X_2_: occupation (farmers/workers = 1, others = 0)
• X_3_: unboiled water consumption
• X_4_: grilled food consumption
• X_5_: tea consumption
• X_6_: shared meals habit
• X_7_: disease duration (>2 years = 1, ≤2 years = 0)
• X_8_: hemoglobin level
• X_9_: albumin level
• X_10_: history of peptic ulcer
• X_11_: family history of tumors

A risk prediction model was then created using the factors that were found. Receiver operating characteristic (ROC) curve analysis was employed to evaluate the prediction model’s diagnostic performance in both the training and validation cohorts. The Hosmer–Lemeshow goodness-of-fit test was used to evaluate the model’s calibration. Statistical significance was defined as a two-sided P value <0.05.

## Result

### Baseline period data

As shown in **Table 1**, several baseline variables differed significantly between the two groups (all P < 0.001), which were then included as independent variables in the multivariate logistic regression model. Compared with the control group, individuals in the H. pylori-positive group had higher proportions of individuals aged ≥50 years (60.00% vs. 45.38%), those engaged in farming or manual labor (57.63% vs. 40.88%), those who frequently consumed unboiled water (79.63% vs. 25.38%), and consumption of grilled foods (74.13% vs. 40.88%). In addition, shared dining habits (65.88% vs. 25.50%), disease duration >2 years (66.00% vs. 32.75%), hemoglobin <120 g/L (39.00% vs. 22.38%), albumin <35 g/L (34.63% vs. 19.13%), previous history of peptic ulcers (25.50% vs. 16.63%), and a family history of tumors (21.63% vs. 6.50%) were all considerably more prevalent in the case group (all *P* < 0.001). These findings suggest that the aforementioned factors are closely associated with H. *pylori* infection. In contrast, no discernible changes were found involving the two groupings in terms of gender, ethnicity, smoking, or alcohol consumption (*P*>0.05; **Table 1**).

### Comparison of GML between the two groups of patients

The pathological changes of GM in the case group were considerably more severe. In the case group, active chronic inflammation (30.38% vs 50.00%), atrophy (35.63% vs 21.75%), intestinal metaplasia (23.25% vs 11.50%), polypoid hyperplasia (29.25% vs 14.63%), dysplasia/intraepithelial neoplasia (32.63% vs 19.25%) The incidence rate was considerably higher than that of the control group (all *P* < 0.001; **Table 2**).

**Table 2:** Comparison of GML in the two groupings of individuals (n/%).

Medication category	Case group (n=800)	Control group (n=800)	*X^2^ value*	*P* value
Active chronic inflammation	243 (30.38)	400 (50.00)	64.091	<0.001
Atrophy	285 (35.63)	174 (21.75)	37.642	<0.001
Intestinal metaplasia	186 (23.25)	92 (11.50)	38.467	<0.001
Polypoid hyperplasia	234 (29.25)	117 (14.63)	49.960	<0.001
Dysplasia/Intraepithelial neoplasia	261 (32.63)	154 (19.25)	37.250	<0.001

### Correlation analysis of H. *pylori* infection and GML

The results of Pearson correlation analysis indicated that H. *pylori* infection was considerably correlated with GML (active chronic inflammation, atrophy, intestinal metaplasia, polypoid hyperplasia, dysplasia, and intraepithelial neoplasia) (r=0.539, 0.401, 0.377, 0.293, 0.545, 0.141; *P* < 0.001).

### Multivariate analysis of Hp infection affecting CG

A multivariate LRA was carried out to determine independent risk factors for H. *pylori* infection in patients with CG. The presence or absence of H. *pylori* infection (infected = 1, not infected = 0) was used as the dependent variable. Variables showing statistically significant differences in **Table 1** were included as independent variables in the regression model.

**Figure 2:**
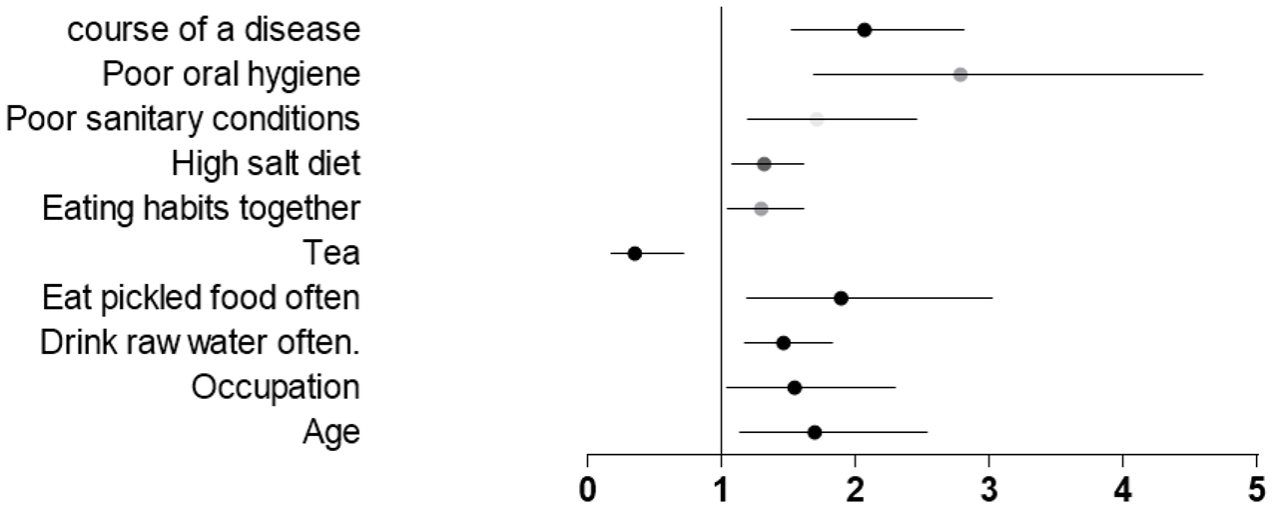
OR forest map.

The multivariate logistic regression analysis identified several independent risk factors for H. *pylori* infection. Age ≥50 years (odds ratio [*OR*]=1.679), occupation as farmers or manual workers (OR = 1.501), frequent consumption of unboiled water (OR = 1.406), consumption of grilled foods (OR = 1.881), habit of sharing meals (OR = 1.319), and disease duration >2 years (OR = 2.044) were all significantly associated with an increased risk of infection (all P<0.05). In contrast, tea consumption was a protective factor against H. *pylori* infection (OR = 0.347, P = 0.001). The results are shown in detail in **Tables 3**, **4**, and **Figure 2**.

**Table 3:** Variable assignment table.

Relevant factors	Variable name	Variable assignment
Age	X_1_	Under 50 years old =0, ≥50 years old =1
Occupation	X_2_	Farmers, workers =1, Cadres and others =0
Frequent consumption of unboiled water	X_3_	yes = 1, no = 0
Frequent consumption of grilled foods	X_4_	yes = 1, no = 0
Tea consumption	X_5_	yes = 1, no = 0
Habit of sharing meals	X_6_	yes = 1, no = 0
Disease course	X_7_	≤2 years =0, More than 2 years =1
Hemoglobin	X_8_	≥120 g/L=0, <120 g/L=1
Albumin level	X_9_	≥35 g/L=0, <35 g/L=1
Previous history of peptic ulcer	X_10_	none=0, have =1
Family history of tumors	X_11_	none =0, have =1

**Table 4:** Multivariate analysis results of *H. pylori* infection affecting CG.

Variable	B	SE	*X^2^*	*P*	OR (95% CI)
Constant term	-0.635	1.028	0.414	0.024	–
Age	0.518	0123	17.736	<0.001	1.679 (1.319~2.136)
Occupation	0.406	0.187	4.714	0.030	1.501 (1.040~2.165)
Frequent consumption of unboiled water (Yes vs. No)	0.341	0.114	8.947	0.003	1.406 (1.125~1.758)
Frequent consumption of grilled foods (Yes vs. No)	0.632	0.219	8.328	0.004	1.881 (1.225~2.890)
Tea consumption (Yes vs. No)	-1.058	0.327	10.468	0.001	0.347 (0.183~0.659)
Habit of sharing meals (Yes vs. No)	0.277	0.106	6.829	0.009	1.319 (1.072~1.624)
Disease course	0.715	0.167	18.331	<0.001	2.044 (1.474~2.836)
Hemoglobin	1.229	0.812	2.291	0.130	3.418 (0.696~16.786)
Albumin level	0.147	0.518	0.081	0.777	1.158 (0.420~3.197)
Previous history of peptic ulcer	1.012	0.624	2.630	0.105	2.751 (0.810~9.347)
Family history of tumors	0.284	0.221	1.651	0.199	1.328 (0.861~2.049)

2.4 Predictive efficiency of prediction model for H.*pylori* infection risk

Based on the 10 indicators with statistically significant differences in the Logistic analysis, a joint prediction model for outcome events was constructed. The formula of the prediction model is as follows: 


$$P\;(\text{Positiveevent})=\frac{1}{1\;+\;e^{(-0.671+0.527\times Age+0.433\times Occupation+0.378\times Drink\;unboiled\;water\;frequently+0.637\times Eat\;grilled\;foods+-1.052\times Drinking\;tea\;+\;0.258\times Eating\;habits\;together+0.725\times Course\;of\;a\;disease)\;}}$$

**Figure 3:**
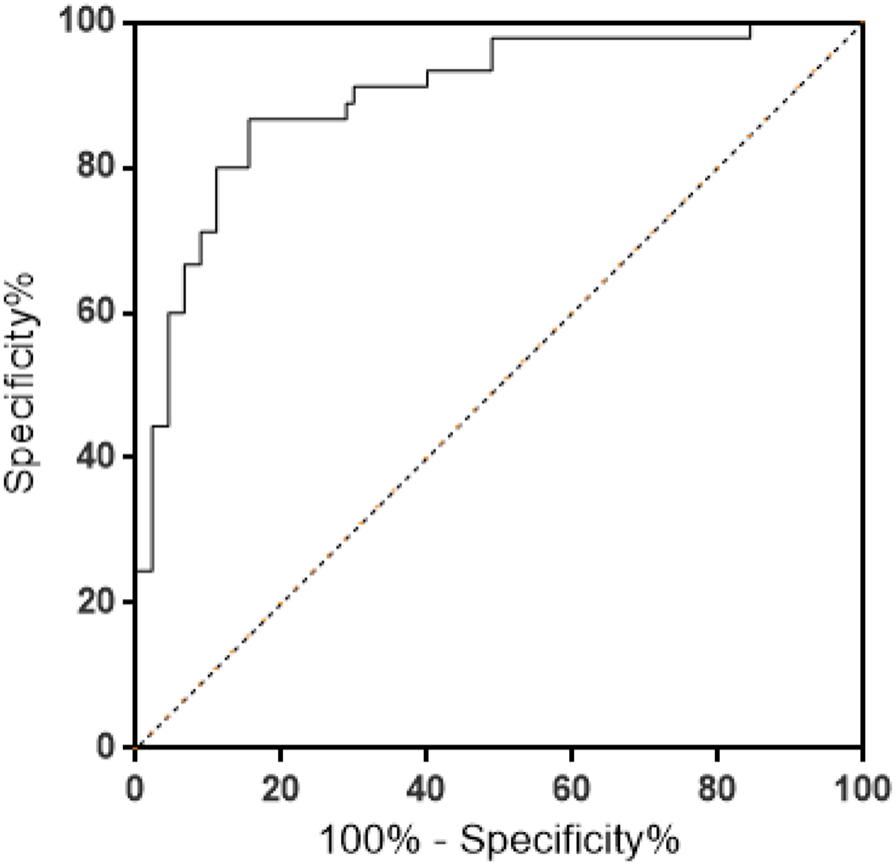
ROC curve of the training set.

**Figure 4:**
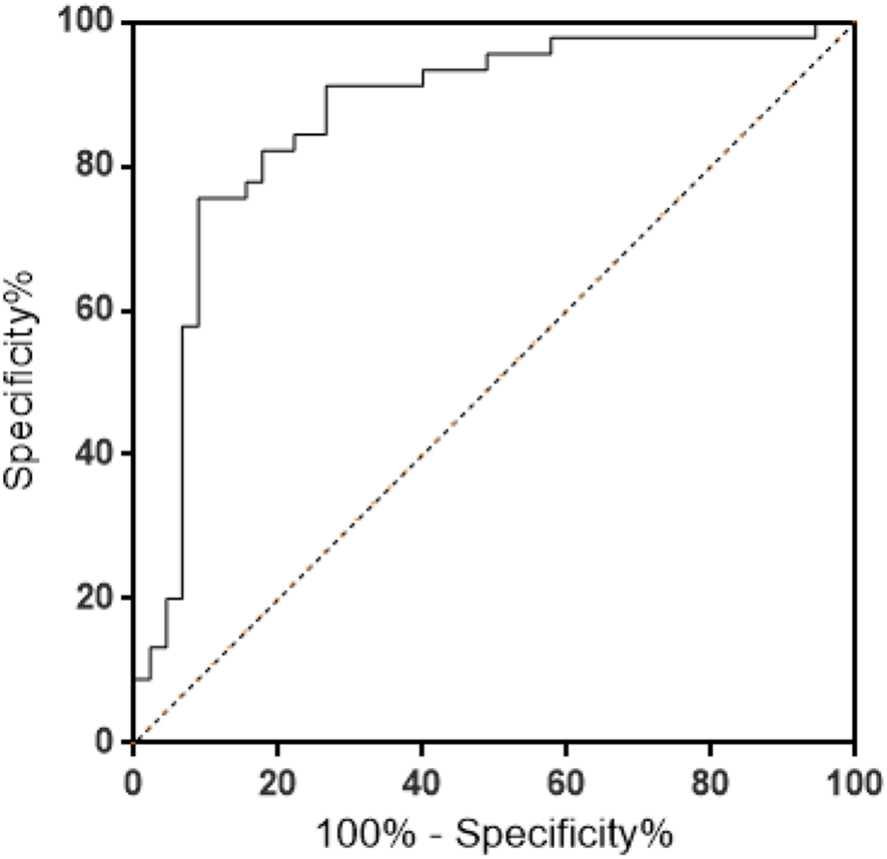
The ROC curve of the validation set.

Using stratified random sampling, the 1,600 participants were divided into a training cohort (n = 1,120) and a validation cohort (n = 480) at a ratio of 7:3. Stratification factors included age (≥50 years vs. <50 years), sex, and H. *pylori* infection status to ensure that baseline characteristics were balanced between the two cohorts. The logistic regression model was developed using the training cohort, and its generalizability was evaluated using the validation cohort. The discriminative performance of the model was assessed using ROC curve analysis. The area under the curve (AUC) was 0.897 (95% CI: 0.832–0.963) for the training cohort and 0.867 (95% CI: 0.787–0.947) for the validation cohort (P < 0.05). The corresponding sensitivities were 88.59% and 82.06%, and specificities were 81.17% and 78.96%, respectively. Additionally, the Hosmer–Lemeshow goodness-of-fit test indicated satisfactory model calibration (χ² = 6.142, P = 0.306). These findings suggest that the constructed model has strong predictive capability for identifying GML associated with H. pylori infection (**Figures 3**, **4**).

## Discussion

H. *pylori* can persist on the gastric mucosal surface for extended periods and is closely associated with a spectrum of GML, including superficial gastritis and gastric ulcers. In recent years, the number of patients with H. *pylori*-related gastric diseases has steadily increased in China (Du et al., 2014; Sharma et al., 2024). Chronic and persistent H. *pylori* infection may lead to progressive atrophic changes in the gastric mucosa, ultimately resulting in CG, peptic ulcers, chronic atrophic gastritis, or even gastric cancer (Malfertheiner et al., 2023). Therefore, early detection and timely intervention for GML are essential to delay disease progression and improve clinical outcomes.

The present study revealed that the incidence of GML—including chronic inflammation, atrophy, intestinal metaplasia, polypoid hyperplasia, dysplasia, and intraepithelial neoplasia—was significantly higher in the case group than in the control group. Pearson correlation analysis demonstrated a notable association between H. *pylori* infection and these lesions, consistent with the findings of most previous studies (Wang et al., 2022; Yu et al., 2022). Among these associations, H. *pylori* infection exhibited the strongest correlations with chronic inflammatory activity and dysplasia, with correlation coefficients exceeding 0.5. This suggests that H. *pylori* infection is a pivotal driver of persistent gastric mucosal inflammation and direct cellular damage or genomic instability. Through its virulence factors, H. *pylori* activates signaling pathways such as NF-κB, inducing substantial infiltration of inflammatory cells (primarily neutrophils and lymphocytes) and perpetuating chronic active inflammation in the lamina propria. Concurrently, H. *pylori*-induced DNA damage and disruption of cell cycle regulation represent critical mechanisms underlying dysplasia development.

In contrast, although the correlation coefficients for H. *pylori* infection with atrophy and intestinal metaplasia also reached statistical significance, they were numerically lower than those observed for active inflammation and dysplasia. This pattern aligns with the pathophysiological progression of H. *pylori*-induced lesions. Gastric mucosal atrophy is generally considered a consequence of prolonged chronic inflammation, particularly active inflammation. Initially, H. *pylori* infection primarily triggers active inflammation; as the infection persists, ongoing inflammatory damage can lead to disruption of glandular structure and a reduction in glandular number, manifesting as atrophy. Intestinal metaplasia, an adaptive or metaplastic change, often occurs against a background of atrophy or coexists with it. It represents a phenotypic transformation of gastric mucosa into intestinal epithelium under sustained chronic injury (including H. *pylori* infection and bile reflux). Thus, atrophy and intestinal metaplasia can be regarded as more advanced or secondary alterations induced by H. *pylori* infection, and their direct association with the infection may be slightly weaker than that of active inflammation and dysplasia—the latter serving as markers of direct damage. Nevertheless, as significant precancerous lesions, their presence indicates that mucosal injury has progressed to a higher-risk stage. Prolonged H. *pylori* infection is associated with chronic active gastritis and precancerous gastric mucosal lesions, including atrophic gastritis and intestinal metaplasia, which are recognized risk factors for gastric carcinogenesis (Hu et al., 2022).

As an inland region in Northwest China, Jiuquan’s unique dietary and lifestyle habits may accelerate the progression of GML. By quantitatively evaluating the correlation between H. *pylori* infection and the severity of gastric mucosal injury, this study elucidates its central role in the pathogenesis of GML. These findings suggest that effective prevention and control of H. *pylori* infection may slow the progression of GML and reduce the long-term risk of gastric cancer.

Furthermore, multivariate LRA identified multiple independent risk factors for H. *pylori* infection. Notably, age ≥50 years emerged as a significant risk factor, which may be attributable to age-related alterations in the physiological structure and function of the gastric mucosa, along with a decline in immune function that increases susceptibility to H. *pylori* colonization. In addition, prolonged exposure to adverse environmental conditions and unhealthy lifestyle habits further exacerbates the infection risk among the elderly population (Tran et al., 2022). Secondly, occupations such as farming or manual labor were significantly associated with an elevated risk of HP infection, likely due to occupational exposure, suboptimal hygiene conditions, and dietary habits. Farmers and manual workers showed a significantly increased risk of H. pylori infection (OR>1), possibly due to environmental exposure and poor hygiene conditions. This occupational group faces a higher likelihood of contact with contaminated water sources, soil, and unsanitary environments. Moreover, occupational stress and irregular work schedules may compromise mucosal resistance and immune defense mechanisms, thereby further increasing infection risk (Demirci et al., 2022; Tran et al., 2022).

Regarding drinking water hygiene, frequent consumption of unboiled water represents a critical risk factor for *H. pylori* infection. Untreated water sources may harbor various pathogens, including *H. pylori*, and direct ingestion significantly elevates infection risk. The persistent habit of drinking unboiled water in certain rural areas of Jiuquan likely serves as a major conduit for local *H. pylori* transmission. Additionally, communal dining—a widespread practice in both social and familial settings across Jiuquan—constitutes a key pathway for *H. pylori* transmission. During shared meals, the bacterium can spread via saliva-contaminated tableware or close interpersonal contact, heightening the risk of cross-infection. In particular, intrafamilial transmission occurs frequently and is considered the primary driver of the persistently high prevalence of *H. pylori* infection in this region (Kayali et al., 2018; Schuppler, 2025).

A disease duration of more than two years was also identified as a significant risk factor for *H. pylori* infection, suggesting that prolonged infection may lead to progressive gastric mucosal damage and an increased risk of lesion development. This finding underscores the importance of early detection and timely treatment in mitigating mucosal injury and preventing disease progression. Tea consumption showed a protective association with H. *pylori* infection (OR<1), which may be attributed to the antibacterial and anti-inflammatory effects of tea polyphenols.

*In vitro* studies have demonstrated that tea polyphenols can inhibit *H. pylori* urease activity and disrupt the integrity of its cell membrane (Deng et al., 2022). However, the precise mechanisms underlying these protective effects *in vivo* remain to be fully elucidated and warrant further investigation through well-designed interventional studies. Moreover, tea drinking may serve as a proxy indicator of a generally healthier lifestyle, including improved dietary hygiene practices such as reduced consumption of untreated water. Therefore, potential confounding factors must be carefully controlled in future studies to validate the independent protective effect of tea consumption against *H. pylori* infection.

Considering the outcomes of the LRA, a risk prediction model was constructed. The model demonstrated robust performance, with relatively high AUC values observed in both the training and validation cohorts. Additionally, the Hosmer–Lemeshow (H–L) goodness-of-fit test indicated satisfactory model calibration. These results suggest that the constructed model may serve as a straightforward and efficient clinical tool for assessing the risk of *H. pylori* infection and associated GML in patients with CG in the Jiuquan region. The model holds potential for facilitating early risk stratification and guiding individualized prevention and intervention strategies.

Nevertheless, this study has several limitations. First, as a retrospective observational study, it is inherently subject to recall bias, particularly regarding self-reported dietary exposures, which may be prone to inaccuracies. Second, *H. pylori* strain typing was not performed; therefore, the association between high-risk virulence factors (such as CagA^+^ and VacA^+^ strains) and the severity of GML could not be assessed. Third, all participants were recruited from a single tertiary hospital, which may limit the generalizability of the findings, especially to underserved rural populations. Future research should incorporate prospective, multicenter cohort studies to enhance representativeness and reduce potential biases. Furthermore, community-based intervention trials targeting the identified risk factors are warranted to evaluate the cost-effectiveness of public health measures, such as expanding access to potable water and promoting individual dining practices.

## Conclusion

In conclusion, this study systematically elucidated the association between *H. pylori* infection and GML in the Jiuquan region and, for the first time, identified region-specific risk factors, including occupational exposure and consumption of unboiled water. Furthermore, the study confirmed the independent protective effect of tea consumption against *H. pylori* infection. These findings offer valuable insights for the targeted prevention and control of gastric cancer in Northwest China. The implementation of a risk prediction model for identifying high-risk populations, along with lifestyle-based interventions, is recommended for integration into regional public health strategies to reduce the burden of *H. pylori*-associated gastric disease.

## Data Availability

The original contributions presented in the study are included in the article/supplementary material. Further inquiries can be directed to the corresponding author.
